# Serial dependence and center bias in heading perception from optic flow

**DOI:** 10.1167/jov.20.10.1

**Published:** 2020-10-01

**Authors:** Qi Sun, Huihui Zhang, David Alais, Li Li

**Affiliations:** Department of Psychology, The University of Hong Kong, Hong Kong SAR; School of Psychology, The University of Sydney, Sydney, Australia; Faculty of Arts and Science, New York University Shanghai, Shanghai, People's Republic of China; NYU-ECNU Institute of Brain and Cognitive Science, New York University Shanghai, Shanghai, People's Republic of China

**Keywords:** heading, serial dependence, optic flow, center bias, self-motion

## Abstract

Previous work shows that observers can use information from optic flow to perceive the direction of self-motion (i.e. heading) and that perceived heading exhibits a bias towards the center of the display (center bias). More recent work shows that the brain is sensitive to serial correlations and the perception of current stimuli can be affected by recently seen stimuli, a phenomenon known as serial dependence. In the current study, we examined whether, apart from center bias, serial dependence could be independently observed in heading judgments and how adding noise to optic flow affected center bias and serial dependence. We found a repulsive serial dependence effect in heading judgments after factoring out center bias in heading responses. The serial effect expands heading estimates away from the previously seen heading to increase overall sensitivity to changes in heading directions. Both the center bias and repulsive serial dependence effects increased with increasing noise in optic flow, and the noise-dependent changes in the serial effect were consistent with an ideal observer model. Our results suggest that the center bias effect is due to a prior of the straight-ahead direction in the Bayesian inference account for heading perception, whereas the repulsive serial dependence is an effect that reduces response errors and has the added utility of counteracting the center bias in heading judgments.

## Introduction

As an observer moves through the environment, light patterns available at the eyes are set in motion as a consequence of head, eye, or body movements. These dynamic motion patterns are known as optic flow (Gibson, 1950), and they contain not only rich information about the world around the observer (including information about the relative distance of objects and their relative motion in the world) but also information about self-movement in the world (Gibson 1950; Longuet-Higgins & Prazdny 1980; Nakayama & Loomis 1974; Royden & Moore, 2012; Warren & Rushton, 2009). For example, one widely studied aspect of optic flow is the invariant it provides about an observer's direction of self-motion, known as heading (Warren & Hannon, 1988). For a constant flow pattern, such as when an observer moves in a straight line, the direction of heading is given by a singular point at the center of the radial flow field known as the focus of expansion (FOE). Observers can use the FOE to determine the direction of heading (Warren, 1976; Warren, Kay, Zosh, Duchon, & Sahuc, 2001; Warren, Morris, & Kalish, 1988), and under optimal conditions, they are able to do so with an accuracy of within 1 degree (Crowell & Banks, 1996; Li, Peli, & Warren, 2002).

One interesting feature of heading perception is that although judgments of heading can be very precise, they also tend to show a bias toward the center of the display where heading is straight ahead (D'Avossa & Kersten, 1996; Li, Peli, & Warren, 2002; Warren & Hannon, 1988; Warren & Kurtz, 1992; Warren & Saunders, 1995). This bias appears to be perceptual and does not depend on the stimulus distribution of presented heading directions (Xing & Saunders, 2016), and is consistent with a Bayesian inference account for heading perception in which a prior for the most frequently encountered heading direction (i.e. straight ahead) biases heading judgments toward it. At the neural level, center bias can be explained by center-weighted spatial pooling (Layton & Fajen, 2017; Warren & Saunders, 1995; Yu, Hou, Spillmann & Gu, 2018). The center bias effect in heading perception effectively compresses heading responses toward the display center/straight ahead.

In addition to center bias, recent work has shown that another bias, serial dependence, occurs commonly in many forms of perception. A serial dependence effect can be attractive or repulsive. An attractive serial dependence effect means that perception of a current stimulus feature is biased toward the value of a previously presented feature. Attractive serial dependence effects occur for a range of stimulus features, including orientation (Cicchini, Mikellidou, & Burr, 2017, 2018; Fischer & Whitney, 2014; Fritsche, Mostert, & de Lange, 2017; Samaha, Switzky, & Postle, 2019), luminance (Fründ, Wichmann, & Macke, 2014), spatial location (Bliss, Sun, & D'Esposito, 2017), numerosity (Fornaciai & Park, 2018), and higher-level features, such as face identity (Liberman, Fischer, & Whitney, 2014) and attractiveness (Taubert, Van der Berg, & Alais, 2016; Xia, Leib, & Whitney, 2016; for a review, please see Kiyonaga, Scimeca, Bliss, & Whitney, 2017). An attractive serial dependence effect tends to reduce the observer's ability to discriminate fine differences in stimuli as small differences are blurred together. In contrast, a repulsive serial dependence effect, such as a negative aftereffect, helps the observer detect small changes in perception because changes around the adapted stimulus produce exaggerated perceptual effects and bias perception away from the previously presented feature. Negative aftereffects are commonly observed in orientation with longer (≥ 5 seconds) stimulus duration (Clifford, Wyatt, Arnold, Smith, & Wenderoth, 2001; Fischer & Whitney, 2014), visual contrast (Georgeson & Harris, 1984; Greenlee & Heitger, 1988), motion speed (Clifford & Wenderoth, 1999), and motion direction (Alais, Verstraten, & Burr, 2005; for a review, see Kohn, 2007).

In the study by Alais, Leung, and Van der Burg (2017), an attractive serial dependence was found for motion direction using simple one-dimensional translation. Using brief motion stimuli that varied in direction from trial to trial, they found that perceived direction on the current trial was biased toward the direction presented on the previous trial. Real world stimuli are usually more complex than this, such as the patterns of optic flow produced when moving through the environment. Accurate perception of the direction of self-motion in the world (i.e. heading) is vital for successful locomotion and navigation with the world, thus improving sensitivity to changes in heading directions through a repulsive dependence may be more important than keeping the continuity of the visual world through an attractive dependence in heading perception.

In this study, we used optic flow stimuli and investigated whether separate from center bias, heading judgments exhibit any serial dependence, and if so, whether the effect would be attractive or repulsive. To separate serial dependence from center bias, we identified and removed the center bias effect in heading responses before testing for the existence of serial dependence in heading perception (Experiment 1). We then varied the reliability of heading information by manipulating the motion coherence level in optic flow to examine how stimulus certainty affects the center bias and serial dependence effects in heading judgments (Experiment 2).

## Experiment 1: Serial dependence in heading perception

In this experiment, we examined whether serial dependence exists in heading perception. To answer this question, on each trial, we presented participants with a display that simulated observer translation through a 3D random-dot cloud. After the presentation, participants were asked to indicate their perceived heading using a mouse-controlled probe. The simulated heading direction changed from trial to trial. We first calculated the errors in heading judgments (i.e. heading errors), defined as the difference between the perceived and the actual simulated heading in each trial. Following previous practice (e.g. Xing & Saunders, 2016), we performed linear regression of the observed heading errors as a function of the presented actual heading to evaluate the center bias effect. If there was a center bias, the perceived heading should be biased toward the display center resulting in an underestimation of heading eccentricity. Accordingly, the observed heading error should be of the opposite sign to the actual heading and the slope of the fitted regression line would be negative.

To evaluate whether separate from the center bias effect, there was any serial dependence in heading judgments. We subtracted the predicted heading error due to center bias from the observed heading error. If there was any serial dependence in heading judgments apart from center bias, it should be revealed in the residual heading error (i.e. the difference between the observed and the predicted heading error). To determine the nature of the serial dependence effect, if any, we performed another linear regression of the residual heading error as a function of relative heading (i.e. the distance in the presented heading direction between the previous and the current trial). For a repulsive serial dependence effect in heading judgments, the perceived heading should be biased away from the heading direction presented in the previous trial, resulting in a negative regression slope. In contrast, for an attractive serial dependence effect, the perceived heading should be biased toward the heading presented in the previous trial, resulting in a positive regression slope.

### Methods

#### Participants

Twenty university students and staff (4 men and 16 women, age: 18–26 years) participated in the study. All were naïve with respect to the purpose of the experiment, and had normal or corrected-to-normal vision. We obtained written informed consent from all participants before the commencement of the experiment. The consent form was approved by the Institutional Review Board at New York University Shanghai.

#### Visual stimuli

**Figure 1.**
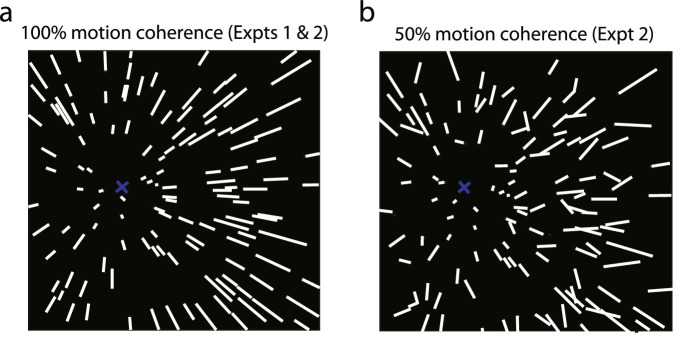
Illustrations of the visual stimuli used in Experiments 1 and 2. (**a**) The instantaneous velocity field of a 3D cloud consisting of 200 white dots (see also Movie 1). (**b**) Fifty percent of the dots in the cloud were replaced with noise dots that moved in random directions while keeping the same speed and duration on the 2D display screen, resulting in 50% motion coherence in optic flow (see also Movie 2). Lines represent the velocity vectors of the dots in the 3D cloud. Blue “x” indicates the heading direction and is not shown in the experimental stimuli.

The display (Figure 1a and Movie 1) simulated an observer translating at 3 m/s through a 3D random dot cloud (80 degrees × 80 degrees, depth range: 0.565 – 2.0 m) that consisted of 200 dots (diameter: 0.6 degrees in visual angle). The direction of the observer translation (heading) was ±32 degrees, ±16 degrees, ±8 degrees, ±4 degrees, ±2 degrees, or 0 degrees. Positive and negative values corresponded to heading to the right or left of the display center, respectively.

The stimuli were generated on an ASUS workstation with a NVIDIA GeForce GTX 970 graphics card at the frame rate of 60 Hz. They were rear-projected on a large screen (101 degrees H × 91 degrees V) with a BENQ projector (native resolution: 1920 × 1080 pixels, refresh rate: 60 Hz). The room was light excluded.

#### Procedure

Participants sat in front of a large screen with their chin stabilized by a chinrest at the viewing distance of 56.5 cm. They viewed the stimuli monocularly with their dominate eye to reduce the conflict between motion parallax (indicating a 3D moving stimulus) and binocular disparity (indicating a flat 2D display screen) depth cues. Before the experiment started, participants’ straight-ahead direction (i.e. their body midline) was aligned with the center of the display screen. A fixation cross appeared at the center of the display, and participants were instructed to fixate on the fixation cross and maintain their eye position there throughout the experiment. On each trial, the simulated self-motion was displayed for 500 ms followed by a blank screen with a horizontal line that appeared across the mid-section of the display. Participants were asked to use a mouse-controlled vertical bar probe to indicate their perceived heading along the horizontal line. The next trial of simulated self-motion started immediately after participants clicked the mouse button.

Each participant completed five blocks of trials, with each block containing 122 trials. The heading directions in the 122 trials were presented using a 11 (heading directions) × 11 (heading directions) matrix. Each cell of the matrix represented a pair of heading directions. The heading directions in a sequence of two trials were determined by randomly selecting a cell from the matrix. If a cell had been chosen, it would be removed from the matrix. The heading directions in the next sequence of two trials thus had to be selected from the remaining cells. This method guaranteed that each of the 11 heading directions (±32 degrees, ±16 degrees, ±8 degrees, ±4 degrees, ±2 degrees, and 0 degrees) was presented both before and after another heading direction once in a block of 122 trials. Participants’ straight-ahead direction (i.e., the display center) was the 0 degree heading direction.

Before the commencement of the experiment, participants were given 20 practice trials to get familiarized with the experiment. No feedback was provided in the practice or experimental trials. The experiment lasted about 30 minutes.

#### Data analysis

To examine the center bias effect in heading perception, for each participant, we first calculated the observed heading error (*HE*), given as:

$$HE=H_{P}-H_{A},$$

where *H_P_* is the perceived heading and *H_A_* is the presented actual heading. A negative heading error indicates the perceived heading is to the left of the presented actual heading and a positive error indicates the opposite. We then performed linear regression of the observed *HE* as a function of the actual heading (*H_A_*):

(1) $$HE=S_{1}\times H_{A}+error,$$

where *S_1_* represents the slope caused by center bias. Specifically, if there is a center bias, the perceived heading should be biased toward the display center resulting in an underestimation of the actual heading with the heading error of the opposite sign to the actual heading (i.e. *S_1_* < 0). The predicted $\tilde{HE}$ due to center bias can thus be estimated from Equation 1.

To examine how the perceived heading is affected by the previously presented heading stimulus (i.e. the serial dependence effect) before factoring out center bias in heading responses, we calculated the relative heading (*H_R_*), which is the distance between the presented heading in the *n^th^* previous trial (*n* = 1, 2, 3, etc.) and the current trial. A negative relative heading indicates the presented heading in the previous trial is to the left of the presented heading in the current trial and a positive relative heading indicates the opposite. We then performed linear regression of the observed *HE* as a function of the relative heading (*H_R_*), given as:

(2) $$HE=S_{2}\times H_{R}+error,$$

where *S_2_* represents the slope caused by the serial dependence effect. Specifically, if *S_2_* is negative, it indicates a repulsive serial dependence effect meaning that the perceived heading is biased away from the previously presented heading, resulting in a heading error opposite in sign to that of the relative heading. In contrast, if *S_2_* is positive, it indicates an attractive serial dependence effect meaning that the perceived heading is biased toward the previously presented heading, resulting in a heading error of the same sign as that of the relative heading.

To examine how the perceived heading is affected by the previously presented heading stimulus (i.e. the serial dependence effect) apart from the center bias effect, we calculated the residual heading error (*HE_R_*) by factoring out the predicted $\tilde{HE}$ due to center bias (see Equation 1) from the observed *HE*, given as:

$$HE_{R}=HE-\tilde{HE.}$$

We then performed linear regression of the residual heading error as a function of the relative heading (*H_R_*), given as:

(3) $$HE_{R}=S_{2}\times H_{R}+error.$$

### Results

**Figure 2.**
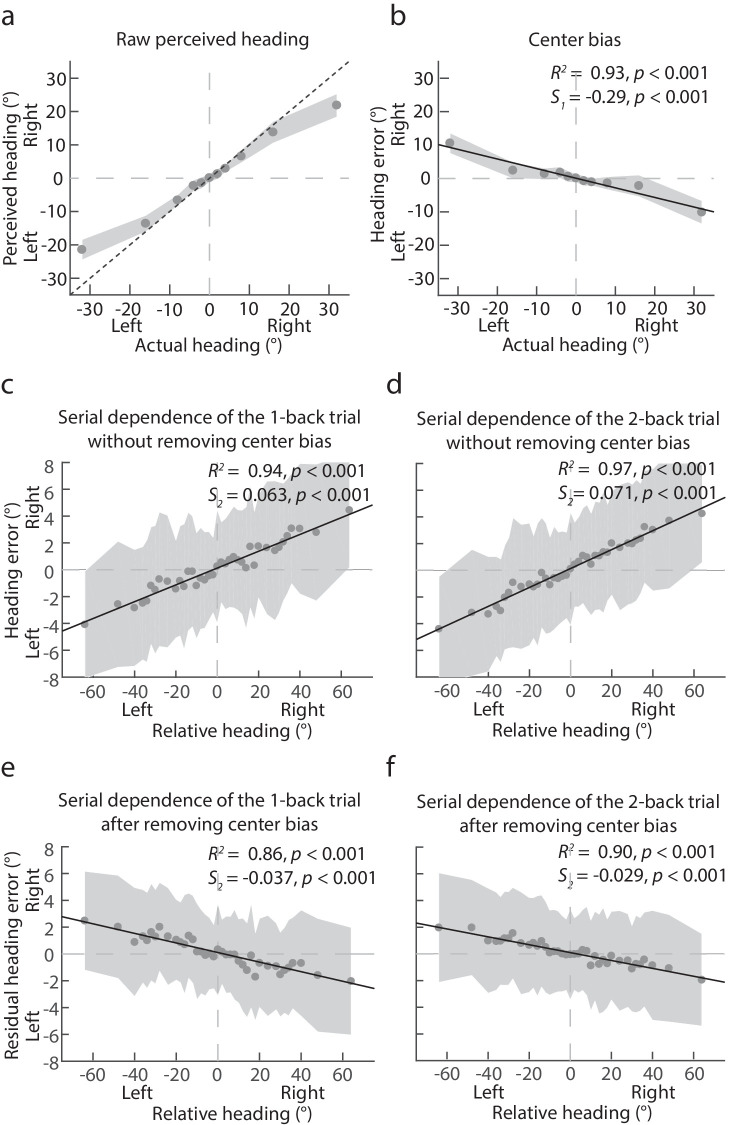
Experiment 1 data. (**a**) Mean perceived heading averaged across participants against actual heading. “Left” and “Right” on the x- and y-axis indicate the actual and the perceived heading to the left or right of the display center, respectively. The dotted line indicates the perfect response. (**b**) Mean observed heading error averaged across participants against actual heading. “Left” and “Right” on the x-axis indicates the actual heading to the left or right of the display center, and “Left” and “Right” on the y-axis indicates the observed heading error to the left or right of the actual heading. (**c**) Mean heading error against relative heading between the first previous trial (i.e. the 1-back trial) and the current trial or (**d**) between the second previous trial (i.e. the 2-back trial) and the current trial. (**e**) Mean residual heading error (i.e. the observed heading error minus the predicted heading error due to center bias) against relative heading between the first previous trial (i.e. the 1-back trial) and the current trial or (**f**) between the second previous trial (i.e. the 2-back trial) and the current trial. “Left” and “Right” on the x-axis indicate that the presented heading of the previous trial was to the left or right of the presented heading of the current trial. “Left” and “Right” on the y-axis indicate that the perceived heading was to the left or right of the predicted perceived heading. The shaded areas indicate ± 1 SD across 20 participants. The black solid lines show the best linear regression fits.

Figure 2a plots the mean perceived heading averaged across participants against the presented actual heading. The perceived heading appears to be biased toward the display center resulting in a systematic underestimation of the actual heading. To evaluate the center bias effect, Figure 2b plots the mean observed heading error averaged across participants against the actual heading. The linear regression analysis with Equation 1 revealed that the fitted line accounted for 93% variance (*p* < 0.001) in the observed heading error. A one-sample *t*-test revealed that the slope (*S_1_*) of the fitted line was significantly below 0 (‒0.29, *t*(9) = ‒11.30, *p* < 0.001, Cohen's *d* = 4.91), indicating a significant center bias effect.

Figure 2c plots the mean heading error averaged across participants against the relative heading between the first  previous (1-back) trial and the current trial and Figure 2d between the second previous (2-back) trial and the current trial. The linear regression analysis with Equation 2 showed that the fitted line accounted for 94% (*p* < 0.001) and 97% (*p* < 0.001) variance in heading error for the 1-back and the 2-back data, respectively. A one-sample *t*-test revealed that the slope (*S_2_*) of the fitted line was significantly larger than zero for both the 1-back (0.063, *t*(37) = 24.15, *p* < 0.001, Cohen's *d* = 3.91) and the 2-back data (0.071, *t*(37) = 35.32, *p* < 0.001, Cohen's *d* = 5.72), indicating a significant attractive serial dependence effect in both cases.

Figure 2e plots the mean residual heading error (i.e. the remaining observed heading error with the predicted heading error due to center bias subtracted) averaged across participants against the relative heading between the first  previous (1-back) trial and the current trial and Figure 2f between the second previous (2-back) trial and the current trial. The linear regression analysis with Equation 3 showed that the fitted line accounted for 86% (*p* < 0.001) and 90% (*p* < 0.001) variance in the residual heading error for the 1-back and the 2-back data, respectively. A one-sample *t*-test revealed that the slope (*S_2_*) of the fitted line was significantly smaller than zero for both the 1-back (‒0.037, *t*(37) = ‒15.35, *p* < 0.001, Cohen's *d* = 4.19) and the 2-back data (‒0.029, *t*(37) = ‒18.17, *p* < 0.001, Cohen's *d* = 3.40), indicating a significant repulsive serial dependence effect in both cases.

To examine how the serial dependence effect varies between the 1-back and 2-back trials, we used a bootstrapping method (see Fischer & Whitney, 2014). Specifically, we generated two distributions of *S_2_* estimates, one for the 1-back data and one for the 2-back data, by bootstrapping the fitting of the linear regression line 10,000 times relying on sampling from participants’ mean error data with replacement on each iteration. This bootstrapping method showed that the mean magnitude of *S_2_* of Equation 2 before factoring out the center bias effect in heading responses was significantly smaller for the 1-back data (mean ± standard deviation [SD]: 0.063 ± 0.0025) than for the 2-back data (0.071 ± 0.0024, *p* = 0.0079). In contrast, the mean magnitude of *S_2_* of Equation 3 after factoring out the center bias effect in heading responses was significantly smaller for the 2-back data (‒0.029 ± 0.0021) than for the 1-back data (‒0.037 ± 0.0022, *p* = 0.005), as is typically found in serial dependence effects in perceptual judgments (e.g. Alais, Leung, & Van der Burg, 2017; Fischer & Whitney, 2014).

### Discussion

The results of this experiment show that, on average, the perceived heading was biased toward the 0 degrees heading direction (i.e. the display center/straight ahead), which caused an underestimation of heading eccentricity. This demonstrates a clear center bias effect in heading judgments that compresses heading responses toward the display center. Before factoring out center bias in heading responses, heading judgments showed an attractive serial dependence (i.e. the heading estimate in the current trial was biased toward the presented actual heading direction in previous trials), indicating that center bias leads to an attractive serial dependence effect in heading perception. After factoring out center bias, heading judgments showed a repulsive serial dependence effect. Furthermore, the attractive serial dependence effect for the 2-back trial showed a significant increase compared with that for the 1-back trial, whereas the repulsive serial dependence effect for the 2-back trial showed a significant reduction compared with that for the 1-back trial, indicating that it decreased with the increase of stimulus history as was commonly observed in previous studies on serial dependence (e.g. Alais, Leung, & Van der Burg, 2017; Fischer & Whitney, 2014).

Based on these results, we argue that the repulsive serial dependence and the center bias effects in heading perception are genuine and distinct processes: they have different signs and their effect sizes show opposite patterns as stimulus history becomes more distant. Because center bias leads to an attractive serial dependence effect, it could reduce the observer's ability to discriminate fine differences in heading, whereas the repulsive serial dependence effect should make the observer more sensitive to small changes in heading. Our finding of a repulsive serial dependence effect in heading perception thus shows that it is a useful effect that helps effectively counteract the negative consequences of the center bias effect in heading perception.

The center bias effect in heading perception is consistent with the Bayesian inference framework that predicts heading estimates should be biased toward straight ahead, the most commonly experienced heading direction in daily life that can be regarded as a prior. If this is true, due to the fact that increasing stimulus uncertainty increases the system's reliance on prior knowledge according to Bayesian theory (Bernardo & Smith, 1994; Cox, 1946; Jaynes, 1986; MacKay, 2003), adding noise to optic flow to increase stimulus uncertainty should increase the center bias effect in heading judgments. It has been reported that serial dependence in orientation judgments increases as stimuli become noisier and less reliable, which can be explained by an ideal observer model where serial dependence reduces reproduction errors and optimizes responses (Cicchini, Mikellidou, & Burr, 2018). If this applies to the repulsive serial dependence effect in heading judgments, we expect that its magnitude should also increase with stimulus uncertainty. In the next experiment, we thus examined how the center bias and serial dependence effects would change with the stimulus uncertainty, and whether the change in the repulsive serial dependence effect could be captured by the ideal observer model in Cicchini et al. (2018).

## Experiment 2: Varying motion coherence level in optic flow

In this experiment, we examined how varying motion coherence in optic flow affected the center bias and serial dependence effects in heading judgments. Similar to the previous studies (e.g. van den Berg, 1992; van den Berg & Brenner, 1994), we manipulated the motion coherence level in optic flow by replacing a proportion of the signal dots in the 3D random dot cloud with randomly moving dots (i.e. noise dots) to reduce motion coherence in optic flow. Given that the precision of heading perception decreases with the decrease of signal-to-noise ratio in motion signals in optic flow (van den Berg, 1992), lowering motion coherence in optic flow increases the uncertainty in the heading stimuli. If center bias is a prior and serial dependence in heading judgments has the effect of optimizing responses (i.e. lowering the deviation from correct response in the presence of sensory noise), both should increase with the decrease of motion coherence in optic flow.

### Methods

#### Participants

Twenty students and staff (5 men and 15 women, age: 19–31 years) from the NYU-ECNU joint research institute participated in the study. All were naïve with respect to the purpose of the experiment, and had normal or corrected-to-normal vision. Two of them participated in Experiment 1. We obtained written informed consent from all participants before the commencement of the experiment. The consent form was approved by the Institutional Review Board at New York University Shanghai.

#### Visual stimuli

The visual stimuli and the experimental setup were the same as in Experiment 1 except that a proportion of the dots (0%, 25%, 50%, or 75%) in the 3D cloud moved in random directions while keeping the same speeds and durations on the 2D display screen (see Figure 1b and Movie 2). Specifically, to generate the noise motion dots, we randomly selected a proportion of the dots in the 3D cloud and calculated their optical motion durations, speeds, and directions (*θ*) on the 2D image screen for each heading direction. We then randomly varied *θ* in the range of 0 degrees < *θ <* 360 degrees on the 2D display screen while keeping their optical motion durations and speeds untouched. This ensured that the global motion amplitude for the four-motion coherence (i.e. signal-to-noise) levels in optic flow (100%, 75%, 50%, and 25%) were equated.

#### Procedure

The experimental setup was the same as in Experiment 1. Each participant in this experiment completed four blocks of trials, with each block containing 122 trials for each motion coherence level so that each of the 11 heading directions (±32 degrees, ±16 degrees, ±8 degrees, ±4 degrees, ±2 degrees, and 0 degrees) was presented before or after another heading direction once. The testing order of motion coherence level was counterbalanced between participants. Before the commencement of the experiment, participants were given 10 practice trials with the optic flow at 100% motion coherence level to get familiarized with the experiment. No feedback was provided in the practice or experimental trials. The experiment lasted about 30 minutes.

#### Data analysis

The data analysis methods used to evaluate the center bias and the serial dependence effects were the same as in Experiment 1.

### Results

**Figure 3.**
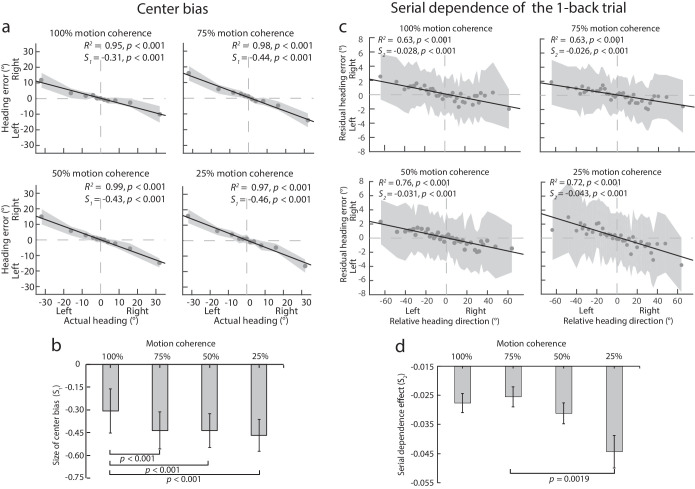
Experiment 2 data. (**a**) Mean observed heading error averaged across participants against actual heading. “Left” and “Right” on the x-axis indicates the actual heading to the left or right of the display center, and “Left” and “Right” on the y-axis indicates the observed heading error to the left or right of the actual heading. The shaded areas indicate ± 1 SD across 20 participants. The black solid lines show the best linear regression fits. (**b**) Mean size of center bias (*S_1_*) averaged across participants as a function of motion coherence level. Error bars are ± 1 SD across 20 participants. (**c**) Mean residual heading error against relative heading between the first previous trial (i.e. the 1-back trial) and the current trial. “Left” and “Right” on the x-axis indicate that the presented heading of the previous trial was to the left or right of the presented heading of the current trial. “Left” and “Right” on the y-axis indicate that the perceived heading was to the left or right of the predicted perceived heading. The shaded areas indicate ± 1 SD across 20 participants. The black solid lines show the best linear regression fits. (**d**) The repulsive serial dependence effect (*S_2_*) as a function of motion coherence level. Error bars are ± 1 SD of the distributions of *S_2_* estimates generated by bootstrapping the fitting of the linear regression line 10,000 times relying on sampling from participants’ mean residual heading error data with replacement on each iteration.

Figure 3a plots the mean observed heading error averaged across participants against the actual heading for each of the four motion coherence levels tested. The linear regression analysis with Equation 1 revealed that the fitted line accounted for 95% or more variance (*p* < 0.001) in the observed heading errors across the four motion coherence levels. A one-sample *t*-test revealed that the slope (*S_1_*) of the fitted line was significantly smaller than 0 for all four motion coherence levels (100% coherence: ‒0.31, *t*(9) = ‒13.29, *p* < 0.001, Cohen's *d* = 5.60; 75% coherence: ‒0.44, *t*(9) = ‒21.08, *p* < 0.001, Cohen's *d* = 8.93; 50% coherence: ‒0.43, *t*(9) = ‒27.05, *p* < 0.001, Cohen's *d* = 11.46; and 25% coherence: ‒0.46, *t*(9) = ‒19.10, *p*< 0.001, Cohen's *d* = 8.15), indicating that the perceived heading was biased toward the display center resulting in an underestimation of heading eccentricity for all four motion coherence levels.

Figure 3b plots the mean center bias effect (*S_1_*) averaged across participants as a function of motion coherence level. A one-way repeated-measures ANOVA revealed a significant main effect of motion coherence (*F*(3, 57) = 19.52, *p* < 0.001, *η^2^* = 0.51). Newman-Keuls tests revealed that the magnitude of the center bias effect for the 100% motion coherence level (mean ± SD: ‒0.31 ± 0.14) was significantly smaller than that for the 25% (‒0.44 ± 0.12, *p* < 0.001), the 50% (‒0.43 ± 0.11, *p* < 0.001), and the 75% motion coherence level (‒0.46 ± 0.11, *p* < 0.001), and the center bias effect was not significantly different from each other for the latter three motion coherence levels (*p* > 0.18).

To evaluate the serial dependence effect, as in Experiment 1, we calculated the residual heading error by removing the predicted heading error caused by center bias from the observed heading error. Figure 3c plots the mean residual heading error averaged across participants against the relative heading between the first previous (1-back) trial and the current trial for each of the four motion coherence levels. The linear regression analysis with Equation 3 showed that the fitted line accounted for 63% or more variance (*p* < 0.001) in the residual heading errors across the four motion coherence levels. A one-sample *t*-test revealed that the slope (*S_2_*) of the fitted line was significantly smaller than zero for all four levels of motion coherence (100%: ‒0.028, *t*(37) = ‒8.12, *p* < 0.001, Cohen's *d* = 1.86; 75%: ‒0.026, *t*(37) = ‒8.18, *p* < 0.001, Cohen's *d* = 1.89; 50%: ‒0.031, *t*(37) = ‒11.09, *p* < 0.001, Cohen's *d* = 2.49; and 25%: ‒0.043, *t*(37) = ‒10.03, *p* < 0.001, Cohen's *d* = 2.26), indicating a significant repulsive serial dependence effect in heading judgments for all four levels of motion coherence in the optic flow. Note that despite the fact that only 122 trials were run for the 100% motion coherence level in the current experiment compared to 610 trials (122 trials × 5 blocks) in Experiment 1, we still found a significant repulsive serial dependence effect in heading judgments.

Figure 3d plots the mean repulsive serial dependence effect (*S_2_*) averaged across participants as a function of motion coherence level. To examine how the repulsive serial dependence effect changed with motion coherence in optic flow, we used the same bootstrapping method as described in Experiment 1 to generate a distribution of *S_2_* estimates for each level of motion coherence. Given that we needed to make six comparisons to determine whether the mean estimate of *S_2_* was statistically significant among the four levels of motion coherence, the significance probability (*p*) was Bonferroni corrected, with *p* = 0.05/6. Although the mean estimate of *S_2_* appeared to increase with motion coherence level, the six comparisons showed that only the mean estimate of *S_2_* for the 75% motion coherence level (mean ± SD: ‒0.026 ± 0.003) was significantly different from that for the 25% motion coherence level (‒0.044 ± 0.006, *p* = 0.0019).

**Figure 4.**
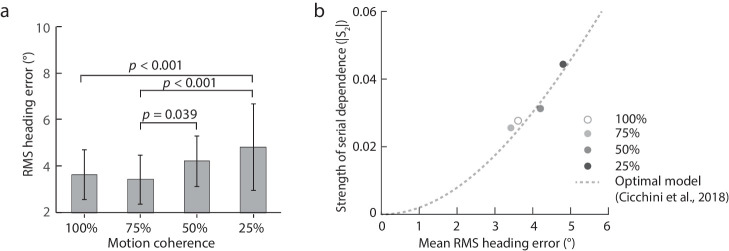
Experiment 2 data. (**a**) Mean RMS heading error averaged across participants against motion coherence. Error bars indicate ± 1 SD across 20 participants. (**b**) Strength of the repulsive serial dependence effect against the mean RMS heading error for the four motion coherence levels in optic flow. The dotted line indicates the predictions of the ideal observer (optimal) model (i.e. Equation 3.7 in Cicchini, Mikellidou, & Burr, 2018, with *d* = 22.3 degrees, the average distance between all relative headings, *σ* = mean RMS error). The model has no free parameters and captures well the relationship between the repulsive serial dependence effect and the mean RMS heading error (*R^2^* = 0.90, *p* < 0.001).

To examine how the precision of heading judgments change with the motion coherence level in optic flow, for each participant, we computed the root mean square (RMS) heading error, which indicates the average vertical distance of the observed heading error from the fitted line with Equation 1. Figure 4a plots mean RMS heading error averaged across participants against motion coherence. A one-way repeated-measures ANOVA revealed a significant main effect of motion coherence (*F*(3,57) = 8.11, *p* = 0.00014, *η^2^* = 0.30). Newman-Keuls tests revealed that the RMS heading error for the 100% (mean ± SD: 3.62 degrees ± 1.07 degrees) and the 75% motion coherence level (3.43 degrees ± 1.05 degrees) was significantly smaller than that for the 25% level (4.81 degrees ± 1.86 degrees; 100%: *p* = 0.00099, 25%: *p* = 0.00037), and the RMS heading error for the 75% level was significantly smaller than that for the 50% level (4.21 degrees ± 1.09, *p* = 0.039), indicating an overall increasing trend of the RMS heading error with decreasing motion coherence in optic flow.

Figure 4b plots the strength of the repulsive serial dependence effect as a function of the mean RMS heading error at each motion coherence level. The dotted line indicates the predictions of the ideal observer model (i.e. Equation 3.7 in Cicchini, Mikellidou, & Burr, 2018) where serial dependence helps reduce response errors, considering both sensory noisiness (measured by the RMS heading error) and inter-stimulus distance (in this case the average distance between all relative headings, *d* = 22.3 degrees). Although there were no free parameters in the model simulation for the change in the repulsive serial dependence effect with the RMS heading error, the model predictions captured the trend of the data very well (*R^2^* = 0.90, *p* < 0.001).

### Discussion

The results of this experiment show that decreasing the motion coherence level in optic flow through increasing the number of motion noise dots increased the center bias effect in heading judgments. That is, with noisier optic flow stimuli, heading judgments were biased more toward the center of the display. This is consistent with the Bayesian inference account for heading perception from optic flow with center bias as a prior.

As expected, adding noise in optic flow increased the RMS heading error reflecting a decrease in the precision of heading judgments. Furthermore, the repulsive serial dependence effect also increased with the motion noise level in optic flow. The change in the repulsive serial dependence effect and the precision in heading judgments with stimulus uncertainty can be well captured by the ideal observer model in Cicchini et al. (2018) where the serial dependence effect minimizes reproduction errors while taking sensory noise and inter-stimulus distance into consideration. This supports the claim that serial dependence in heading judgments has the effect on reducing reproduction errors and thus optimizing responses.

Note that the ideal observer model in Cicchini et al. (2018) is built to account for the attractive serial dependence effect in orientation judgements. It predicts the current orientation estimate is a weighted average of previous and current orientations based on their relative reliability and distance, which can reduce the variance in orientation judgments at the cost of inducing a bias. Here, the change in the repulsive serial dependence and in the precision of heading judgments with stimulus uncertainty could also be well captured by this model, suggesting a common computational mechanism underlying serial dependence, irrespective of the sign of the effect.

## General discussion

Combining the results from the two experiments, we have found that separate from the previously reported center bias effect that compresses heading responses toward the display center/straight ahead, heading perception also shows a repulsive serial dependence effect. That is, after factoring out center bias in heading responses, the current heading estimate is biased away from previously seen heading directions, resulting in a repulsive serial dependence effect in heading judgments, which would help an observer detect small changes in heading directions by perceptually expanding stimulus differences. Before factoring out center bias in heading judgments, the current heading estimate is biased toward previously seen heading directions, thus exhibiting an attractive serial dependence effect that would decrease the observer's ability to discriminate fine stimulus differences by effectively reducing perceptual differences between stimuli. Our findings therefore suggest that the repulsive serial dependence is a useful effect in counteracting the center bias effect in heading perception to increase the visual system's overall sensitivity to changes in heading.

Another important finding of the current study is that both the center bias and serial dependence effects in heading judgments are affected by the motion coherence level in optic flow. Specifically, decreasing motion coherence in optic flow increases the strength of both the center bias and serial dependence effects. The increase of the center bias effect with noisy optic flow is consistent with the idea that the center bias is due to prior knowledge that favors the center straight-ahead direction, and that heading perception is a performance-optimizing Bayesian process (see also Fetsch, Turner, DeAngelis, & Angelaki, 2009). This is because, according to Bayesian theory, as stimulus uncertainty increases, the brain increasingly relies on prior knowledge to make responses (Bernardo & Smith, 1994; Cox, 1946; Jaynes, 1986; MacKay, 2003). This trade-off has been illustrated for not only a variety of visual processing (e.g. Bülthoff, 1991; Kersten & Yuille 2003; Mamassian & Landy, 2001; van Ee, Adams, & Mamassian, 2003; Weiss, Simoncelli, & Adelson, 2002,) but also sensorimotor learning (Körding & Wolpert, 2004; Tassinari, Hudson, & Landy, 2006), path integration (Petzschner & Glasauer, 2011), and sensitivity to timing formation (Jazayeri & Shadlen, 2010).

The increase of the repulsive serial dependence effect with decreasing motion coherence in optic flow agrees with existing models of serial dependence. It has been proposed that serial dependence effects help increase the continuity and reliability of current perception by integrating current and recently seen stimuli (Fischer & Whitney 2014). Models based on the Kalman filter show that the weight given to the previous stimulus depends on sensory noisiness that is affected by stimulus reliability (Cicchini, Mikellidou, & Burr, 2018). Here, we reduced stimulus reliability by reducing motion coherence in optic flow, which reduced the precision of heading judgments but produced stronger serial dependence effects. The increase in the strength of the serial dependence effect with the decrease of the precision of heading judgments as stimulus uncertainty increases is well predicted by the ideal observer model in Cicchini et al. (2018) (see Figure 4). This is consistent with previous studies that have used various approaches to reduce stimulus reliability to affect serial dependence, including adding noise to grating stimuli or lowering their contrast, spatial frequency or spatial extent (Cicchini, Mikellidou, & Burr, 2018; Fischer & Whitney 2014). Together, these findings suggest that stimulus uncertainty is a major driver of serial dependence, regardless of the source of noise, and the brain is able to use serial dependence to reduce reproduction errors thus optimize responses.

Note that we conducted a simple linear regression analysis on the observed heading errors (Equation 1) and then another simple linear regression analysis on the residual heading errors (Equation 3) to reveal the repulsive serial dependence effect in heading judgments. To confirm the findings of the current study, we also conducted a multiple linear regression analysis on the observed heading errors with both center bias and serial dependence terms (Equation 5) and found the multiple regression model accounted for significantly more variance in the observed heading error data than did the simple regression model with only the center bias term (Equation 4) for all our experimental conditions (see Appendix for details). In addition, the fitted center bias and serial dependence slopes with Equation 5 followed the same pattern of the reported slopes in the Results sections above. This supports the validity of the two-step simple linear regression analysis we performed, which helps clarify the logic and clearly visualize both effects in heading judgments.

Most previous studies on serial dependence have found attractive serial dependence effects (see Kiyonaga, Scimeca, Bliss, & Whitney, 2017 for a review). Some studies (Bliss, Sun, & D'Esposito, 2017; Fritsche, Mostert, & de Lange, 2017; Samaha, Switzky, & Postle, 2019) tested a large stimulus range and found an attractive serial dependence effect for relatively small inter-stimulus distances but a repulsive serial effect for large distances (e.g. > 60 degrees in orientation judgments). Taubert, Alais, and Burr (2016) found serial dependence effects that were attractive for gender but repulsive for facial expressions. They proposed that repulsive serial dependences are more likely to occur for dynamic attributes (such as facial expressions) because the visual system gains more from contrastive adaption to maximize its sensitivity to change. In contrast, attractive serial dependences are more likely to occur for stable attributes (such as gender) because they can be safely integrated over time. The repulsive serial dependence effect in heading judgments found in the current study supports this proposal. Although using simple one-dimensional motion translation stimuli, Alais et al. (2017) found an attractive serial dependence effect in motion direction judgments, optic flow stimuli are more complex and dynamic. Accurate perception of heading is also important for successful navigation and survival, thus improving sensitivity to change in heading through a repulsive serial dependence effect would be more important than maintaining continuity of the visual world through an attractive dependence.

Previous work has suggested that the serial dependence effect is perceptual that occurs independently of decision by showing that the current response can be biased toward the previously seen stimuli even when no response was made in the previous trials (e.g. Fischer & Whitney, 2014). Nevertheless, the fact that serial dependence effects increase in strength with the time delay between stimulus presentation and response generation suggests that the serial effect occurs at a post-perceptual stage, possibly in working memory (Fritsche, Mostert, & de Lange, 2017). More recent work proposes that serial dependences occur at both perceptual and decision stages (Cicchini, Mikellidou, & Burr, 2018). Although the findings of the current study do not speak to this question, given that heading perception requires the integration of both visual and vestibular information (e.g. Fetsch, Turner, DeAngelis, & Angelaki, 2009; Gu, Cheng, Yang, DeAngelis, & Angelaki, 2016; Yang & Gu, 2017), and the cortical areas receiving vestibular information are also involved in decision making (e.g. Harvey, Coen, & Tank, 2012), it is likely that the repulsive serial dependence effect in heading judgments also occurs at both perceptual and decision stages.

In summary, in the current study, we managed to separate center bias and serial dependence effects in heading judgments. We found that both effects influence heading perception. The center bias effect compresses heading responses toward the center straight-ahead direction and increases with stimulus uncertainty. This supports the idea that heading perception follows the Bayesian inference framework and center bias is due to prior knowledge that favors the straight-ahead direction. An interesting new finding of the current study is the repulsive serial dependence effect in heading judgments, which in general helps the detection of small stimulus changes. This repulsive serial effect also increases with stimulus uncertainty, following the prediction of the ideal observer model in Cicchini et al. (2018) where serial dependence helps reduce reproduction errors thus optimize responses. Together, the findings of the current study support the claim that there is a repulsive serial dependence effect in heading perception, in addition to the well-known center bias, and that the brain can use the repulsive serial dependence effect to optimize responses and to counteract the center bias in heading judgments.

## Supplementary Material

Supplement 1

Supplement 2

## Appendix

### Multiple linear regression analysis

We carried out a multiple linear regression analysis to examine whether having two variables (one for center bias and the other for serial dependence) in a linear regression model would significantly explain more variance in the observed heading error data than having only one variable (for center bias) in the model. Specifically, similar to Equation 1, we first fitted the observed heading errors (*HE*) with a linear function of the actual heading (*H_A_*), given as:

(4) $$HE=S_{1}^{'}H_{A}+error,$$

where *S_1_’* represents the slope caused by center bias. Then, we conducted multiple linear regression of the observed *HE* as a function of both actual heading (*H_A_*) and relative heading (*H_R_*, i.e. the distance in the presented heading between the previous and the current trial), given as:

(5) $$HE=S_{1}^{'}H_{A}+S_{2}^{'}H_{R}+error,$$

where *S_2_’* represents the slope due to serial dependence. Again, a negative *S_2_’* indicates a repulsive serial dependence effect meaning that the perceived heading is biased away from the previously presented heading, resulting in the sign of the residual heading error opposite to that of the relative heading. In contrast, a positive *S_2_’* indicates an attractive serial dependence effect meaning that the perceived heading is biased toward the previously presented heading, resulting in the sign of the residual heading error the same as that of the relative heading.

When fitting the linear regression model to the data, we used the least square method given as:

(6) $$MSE=\frac{1}{N}\sum_{i=1}^{N}{\left(HE_{i}-{\tilde{HE}}_{i}\right)}^{2},$$

where MSE is the minimum squared error, *HE_i_* and ${\tilde{HE}}_{i}$ are the actual and predicted heading errors of *i*^th^ data point, *N* is the size of the data set. For Experiment 1, *N* = 12,100 (20 participants × 5 blocks × 121 relative headings); for Experiment 2, *N* = 2,420 (20 participants × 121 relative headings) for each motion coherence level.

Because Equations 4 and 5 show two nested regression models, we performed an ANOVA for regression (i.e. *F*-test) to determine whether the complex model (Equation 5) is better than the simple version of the same model (Equation 4) in explaining the variance of the observed heading error data. The results of the model fitting as well as the *F*-tests are listed in Table A1. In summary, Equation 5 with the extra term of serial dependence explained significantly more variance (although small) than did Equation 4 with only the term of center bias. In addition, both fitted parameters *S_1_’* and *S_2_’* for the complex model (Equation 5) followed the same pattern of the fitted parameters *S_1_* and *S_2_* in the Results sections above, indicating the consistency between the multiple regression analysis and the two-step linear regression analysis as described in the main text.

**Table A1.** The results of the linear regression analyses with Equations 4 and 5. ***: *p* < 0.001, **: *p* < 0.01, *: *p* < 0.05.

Condition	Model	*S_1_’*	*S_2_’*	Adjusted *R^2^*	*F* value
Experiment 1	*Eq 4*	−0.20***		0.308	38.56***
	*Eq 5*	−0.22***	−0.017***	0.311	
Experiment 2 100%	*Eq 4*	−0.20***		0.405	12.62***
	*Eq 5*	−0.19***	−0.010*	0.411	
Experiment 2 75%	*Eq 4*	−0.33***		0.648	18.85***
	*Eq 5*	−0.31***	−0.0098*	0.651	
Experiment 2 50%	*Eq 4*	−0.37***		0.575	4.03*
	*Eq 5*	−0.38***	−0.013*	0.576	
Experiment 2 25%	*Eq 4*	−0.38***		0.525	9.90**
	*Eq 5*	−0.37***	−0.014*	0.527	
